# A Prototypical First-Generation Electronic Cigarette Does Not Reduce Reports of Tobacco Urges or Withdrawal Symptoms among Cigarette Smokers

**DOI:** 10.1155/2017/6748948

**Published:** 2017-03-26

**Authors:** Arit M. Harvanko, Catherine A. Martin, Richard J. Kryscio, William W. Stoops, Joshua A. Lile, Thomas H. Kelly

**Affiliations:** ^1^Department of Psychology, University of Kentucky, Lexington, KY, USA; ^2^Department of Psychiatry, University of Kentucky, Lexington, KY, USA; ^3^Department of Biostatistics, University of Kentucky, Lexington, KY, USA; ^4^Department of Behavioral Science, University of Kentucky, Lexington, KY, USA

## Abstract

It is unknown whether first-generation electronic cigarettes reduce smoking urges and withdrawal symptoms following a 24 h deprivation period. This study tested whether a first-generation electronic cigarette reduces smoking urges and withdrawal symptoms in cigarette smokers. Following 24 h of tobacco deprivation, using a within-subjects design, eight nontreatment seeking tobacco cigarette smokers (3 females) administered 10 puffs from a conventional cigarette or a first-generation electronic cigarette containing liquid with 0, 8 or 16 mg/ml nicotine. Conventional cigarettes ameliorated smoking urges and electronic cigarettes did not, regardless of nicotine concentration. First-generation electronic cigarettes may not effectively substitute for conventional cigarettes in reducing smoking urges, regardless of nicotine concentration.

## 1. Introduction

Electronic cigarettes (ECs) are battery-powered devices that use a heated coil to aerosolize liquids typically containing nicotine. At least three separable categories of ECs have been recognized: first-, second-, and third-generation. First-generation ECs have relatively low-capacity batteries, typically nonrefillable liquid cartridges, and few (if any) variable settings, operate at lower wattages, and are activated when the user inhales. Second-generation devices typically have larger rechargeable batteries, refillable liquid tanks, and some adjustable parameters (e.g., variable voltage) that allow the users to tailor the function of the device and are activated by pressing a button as a user inhales. Third-generation devices are similar to second-generation devices with the addition of a greater number of user-customizable parameters (e.g., voltage or wattage) and configurations (e.g., different types of tanks or batteries). Recent survey data suggest that tobacco cigarette smokers often begin using first-generation ECs and then later transition to 2nd- or 3rd-generation devices [1]. Tobacco smokers initiate EC use with a first-generation device because they more closely resemble tobacco cigarettes compared to 2nd- and 3rd-generation devices [1]. It is also possible that smokers are attracted to them because of their lower cost and wider availability compared to later generation devices. Since many begin EC use with a first-generation EC, initial experiences with first-generation ECs likely influence the probability to continue use of ECs.

Previous studies suggest that first-generation ECs containing liquids with 16–18 mg/ml nicotine concentrations, and to a lesser extent a 0 mg/ml nicotine concentration, ameliorate some smoking urges and withdrawal symptoms [2, 3]. Yet, those studies have used tobacco deprivation periods of 12 h or less prior to administration of an EC [2–5]. Further, none of the aforementioned studies collected baseline measures of smoking urges and withdrawal symptoms prior to tobacco deprivation and are unable to differentiate symptom alleviation from other subjective effects following EC self-administration. Thus, there has not yet been a rigorous examination of alleviation of smoking urges and withdrawal symptoms by first-generation ECs following 24 h tobacco deprivation. The present study compared the effects of a prototypical first-generation EC using three nicotine concentrations (0, 8, and 16 mg/ml) on smoking urges and withdrawal symptoms following 24 h of smoking deprivation; a conventional tobacco cigarette (CC) was also tested as a positive control and comparator for the EC conditions.

## 2. Materials and Methods

### 2.1. Participants

Participants were recruited from the community using online advertisements. Respondents completed medical and psychological screening questionnaires, a one-year timeline follow-back for CC use, the Fagerstrom Test for Nicotine Dependence, and a questionnaire assessing lifetime quantity and frequency of EC use. Inclusion criteria were smoking an average of 10 or more nonmenthol tobacco cigarettes per day for at least one year, breath carbon monoxide (CO) levels of 10 ppm or higher during screening, and not being a regular EC user (defined as daily use of an EC for the past seven days). Individuals were excluded if they expressed interest or engagement in smoking cessation treatment. The University of Kentucky Medical Institutional Review Board approved this study.

A total of 9 individuals (3 females), between 22 and 47 years of age, met eligibility criteria and started the study. Eight completed the study; one female participant failed to meet the tobacco deprivation breath CO criterion needed to begin the study on two occasions and then was lost to follow-up. All participants identified their race as Caucasian.

### 2.2. Design

A placebo-controlled, double-blind, randomized, within-subject design was used to examine the behavioral effects of controlled puffs from a CC (participant's own brand) and EC producing aerosol from commercial solutions containing 0, 8, or 16 mg/ml of nicotine following 24 h of smoking deprivation.

### 2.3. Schedule

The study consisted of one 4 h practice session followed by four two-session test blocks; a minimum of 48 h separated each test block. Based on convenience, participants chose session start times that remained constant throughout their participation in the study.

The practice session was designed to familiarize participants with how to use the EC [4]. Following 24 h of tobacco deprivation, participants administered six puffing bouts from an EC with 16 mg/ml nicotine concentration, with each bout consisting of 10 puffs. The six bouts were separated by 30 min.

Test blocks were designed to measure the effects of CC and EC use following 24 h of smoking deprivation. Each test block consisted of a “baseline” session, which was not associated with any restrictions on tobacco smoking, directly followed by 24 h of tobacco deprivation, and then a “deprivation” session. During each session, a behavioral assessment was completed before and after administration of a puff bout consisting of 10 two-second puffs, each separated by 30 seconds. During the “baseline” session, puff bouts were always administered from a CC. A puff bout from either an EC with one of three nicotine concentrations (0, 8, and 16 mg/ml) or a CC was administered in random order during the “deprivation” sessions. The paced puffing procedure was utilized for administering puff bouts in order to maintain standardized puffing topographies across all test conditions, and adherence to the paced puffing procedure was verified by staff members using camera images of participants smoking, and inhalation data displays from a volumetric transducer. During the four test blocks the effects of EC, with one of three nicotine concentrations (0, 8, and 16 mg/ml), or a CC, were tested in a randomized order.

Compliance with the 24 h tobacco deprivation conditions prior to the practice session and each of the four “deprivation” sessions during test blocks was verified using breath CO criteria of ≤6 ppm or 10% of predeprivation breath CO, whichever was greater. Sessions were rescheduled if participants failed to meet the breath CO criteria. On two occasions this occurred within test blocks; participants restarted the two-day block (i.e., repeated the “baseline” session) on each occasion.

### 2.4. Assessments

To assess smoking withdrawal symptoms the Minnesota Nicotine Withdrawal Scale (MNWS) [6] was used. The Questionnaire of Smoking Urges-Brief (QSU-B) [7] was used to assess smoking urges, and the Visual Analog Scale-Smoking Effects (VAS-SE) [8] and Visual Analog Scale-Postsmoking (VAS-PS) assessed smoking or EC use effects. Cognitive tasks included the Digit Symbol Substitution Task (DSST) [9] and the Rapid Information Processing Task (RIP) [10]. Heart rate and blood pressure were also recorded.

### 2.5. Drug

A Blu® cartomizer with “Classic Tobacco” liquid flavoring (Lorillard Technologies, Inc., Greensboro, NC), a first-generation EC widely available throughout the United States, served as the EC (webpage for this device archived at http://www.webcitation.org/6nuZa31ga). Cartomizers were all purchased simultaneously at the beginning of the study in order to minimize possible variations in batches. Cartomizers offered by the manufacturer with the labels “no nicotine” (0 mg/ml), “low nicotine” (6–8 mg/ml), and “high nicotine” (14–16 mg/ml) were used to experimentally manipulate nicotine concentrations. Cartomizer labels were covered from study participants and investigators working on this study to maintain the double-blinded design. Nonmenthol commercially available own brand cigarettes (i.e., participants preferred brand) were supplied for the CC conditions.

### 2.6. Data Analysis

To identify tobacco deprivation effects on withdrawal symptoms and smoking urges, presmoking assessments during “baseline” and “deprivation” sessions were compared. To determine if EC or CC use altered smoking urges and withdrawal symptoms, reports of withdrawal symptoms and smoking urges were compared prior to, and following, EC or CC administration during deprivation sessions. Separate mixed models were used to test for effects between each time point, with dose condition as a variable only for models examining change in smoking urges and withdrawal symptoms before and after CC or EC use during deprivation sessions. When significant effects of dose condition were found, post hoc analyses were conducted using* t*-tests to examine differences between least-square means of each dose condition. Hochberg's step-up procedure [11] was used to control error rates for each family of pairwise comparisons. Mixed models were fit using PROC Mixed in the SAS statistical software package, version 9.3 (SAS Institute Inc., Cary, NC).

## 3. Results and Discussion

### 3.1. Results

Six of eight participants had previously used an EC, with an average of 27 days (SE = 13) of lifetime use, and last use occurring an average of 256 days (SE = 142) prior to screening. Participants reported smoking an average of 20 (SE = 3.3) cigarettes per day and scored an average of 5.6 (SE = .8) on the Fagerstrom Test for Nicotine Dependence. Minimal alcohol (*M* = .8, SE = .3 days/week) and marijuana (*M* = .9, SE = .6 occasions/month) use was reported. Breath CO levels decreased from an average of 29.0 ppm (SE = 5.9) prior to smoking on “baseline” sessions to an average of 5.5 ppm (SE = .4) prior to smoking during “deprivation” sessions.

Statistically significant (*p*s < .05) withdrawal symptoms were observed on seven of thirteen MNWS items and heart rate (“baseline versus 24-Dep” column in Table 1). There were no significant withdrawal effects on cognitive tasks (DSST and RIP) or VAS measures of smoking effects.

**Figure 1:**
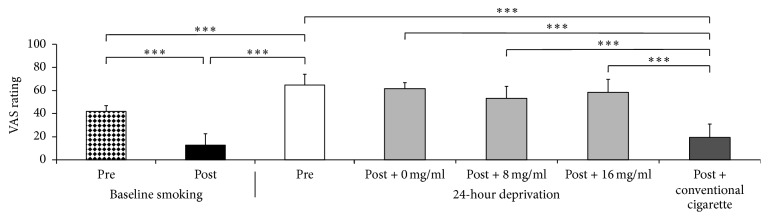
Self-report of “I have a desire for a cigarette right now.” Subjective ratings of “I have a desire for a cigarette right now” on a 100-point Visual Analog Scale. A significant decrease was observed following baseline session smoking and a significant increase was observed following 24 h tobacco deprivation. Ratings prior to CC (own brand) or EC use during deprivation sessions were also significantly higher than presmoking ratings during baseline sessions. Following cigarette administration during deprivation sessions, CC engendered significantly lower ratings compared to EC dispensing aerosol from 0, 8, or 16 mg/ml nicotine concentrations.  ^*∗∗∗*^*p* < .001.

Mixed models indicated a significant effect of drug on “desire or craving to smoke” on the MNWS (*F*[3,21] = 3.29,* p *= .041), six of nine measures on the QSU-B (*p*s < .05), and heart rate (*F*[3,21] = 3.45,* p* = .035) following CC or EC use on deprivation sessions. Following CC use, post hoc estimates of least-square means indicated a significant decrease of “desire or craving to smoke” on the MNWS, six of nine items on the QSU-B (e.g., “I have a desire for a cigarette right now,” “I am going to smoke as soon as possible”), and a significant increase in heart rate (least-square means of these changes shown in Table 1). A prototypical example of the pattern of effects on subjective measures is indicated on the QSU-B item “I have a desire for a cigarette right now,” shown in Figure 1. No significant reductions in smoking urges were indicated following use of the EC at any nicotine concentration.

The mixed model for change in total trials on the DSST from before to after CC or EC use during deprivation sessions indicated a significant effect of drug (*F*[3,21] = 3.18,* p* = .045). Post hoc estimates of least-square means indicated significant increases in total trials on the DSST following CC and 8 mg/ml EC (Table 1). The mixed model for rating of “Lightheaded” on the VAS-SE from before to after CC or EC use during deprivation sessions indicated a significant effect of drug (*F*[3,21] = 8.90,* p* < .001). Post hoc estimates of least-square means indicated a significant increase in rating of “Lightheaded” on the VAS-SE following CC, but not ECs (Table 1). Neither of these measures was altered by tobacco deprivation (Table 1). The VAS-PS was only administered following EC or CC use and, compared to CC, significantly lower ratings of “did you like the effects?” were observed following use of EC at all nicotine doses (*t*[21] = 2.89 to 3.56,* p* < .001) and “did you enjoy the cigarette?” (*t*[21] = 3.40,* p* = .003) and “do you feel stimulated?” (*t*[21] = 3.50,* p* = .002) following the 0 mg/ml EC condition; significantly higher ratings of “do you want to smoke again?” were observed following use of EC at all doses (*t*[21] = 4.19 to 5.55,* p* < .001).

### 3.2. Discussion

This study examined the subjective and physiological effects of a commercially available first-generation EC delivering aerosol from experimentally manipulated nicotine concentrations compared to preferred brand CCs among regular tobacco smokers following 24 h of tobacco deprivation. Reports of withdrawal symptoms and increases in smoking urges were observed on the MNWS, and QSU-B, and on heart rate following 24 h of tobacco deprivation. Smoking CCs after 24 h of tobacco deprivation ameliorated the increase in smoking urges and physiological withdrawal symptoms (i.e., heart rate), while no attenuation of these effects followed use of ECs, regardless of nicotine concentration. An isolated effect of 8 mg/ml EC was observed on DSST trial rate; since DSST performance was not altered by tobacco deprivation, this would not be considered a tobacco withdrawal alleviation effect.

Self-reported smoking urges and withdrawal effects were consistent with previous research [7, 8], and reports of “desire or craving to smoke” on the MNWS and six QSU-B items (e.g., “I have a desire for a cigarette right now,” Figure 1) measured during deprivation sessions significantly decreased following smoking CCs but not ECs, suggesting that first-generation ECs may be less effective than CCs in managing self-reported smoking urges. This is consistent with previous studies comparing CCs to ECs delivering aerosol from a single nicotine concentration [2, 3, 12]. Unlike previous studies [2–5], however, the first-generation EC tested here failed to attenuate any smoking urges. There are several possible explanations for the failure of a first-generation EC to attenuate smoking urges following 24 h tobacco deprivation in this study. First, previous studies did not differentiate whether or not EC effects were associated with the alleviation of verified tobacco deprivation effects (i.e., symptoms measured pre- and postsmoking deprivation). The current study, however, used a baseline session prior to testing each EC and CC condition in order to focus only on verified tobacco deprivation effects. This difference likely made the current study relatively more conservative in terms of which variables were considered “withdrawal symptoms.” Second, the 24 h deprivation period used in the current study might have engendered larger deprivation effects that were insensitive to use of the first-generation ECs. Third, two of the aforementioned studies used ad-lib puffing periods from ECs with 16 or 18 mg/ml nicotine concentrations [2, 5], suggesting that allowing the user to control the timing and number of puffs from an EC might more effectively ameliorate withdrawal symptoms. Fourth, the aforementioned studies did not use Blu ECs, and these devices might not be as effective at delivering nicotine as first-generation ECs used in other studies. It is also worth noting that newer ECs that are more powerful (greater wattage) or contain higher nicotine concentrations might be more effective at delivering nicotine and reducing tobacco urges and withdrawal symptoms compared to the device used in this study. Fifth, although previous research has indicated that the nicotine concentration in at least one commercially obtained Blu EC cartomizer was accurate [13], the reliability of the packaging label is not known, nor is the concentration of nicotine delivered in aerosol. Sixth, six of eight participants in this study had reported having discontinued use of an EC, with minimal prior EC use (*m* = 27 days since last use). The fact that these six participants had discontinued use of an EC and that two participants had never attempted to use an EC previously may indicate that the sample used in this study is biased towards individuals who are less likely to respond to ECs. Lastly, although procedures demonstrated to be effective in training participants to extract nicotine from first-generation devices were used here [4], nicotine delivery was not assessed biologically and the absence of effect on tobacco withdrawal symptoms might be due to ineffective nicotine delivery. In conclusion, this type of EC may not be effective at reducing tobacco urges when used under similar conditions as the current study.

## Figures and Tables

**Table 1:** Least-square mean changes in subjective, cognitive, and physiological effects by session and condition.

Variable	Baseline versus 24-Dep.	Conventional cigarette	Electronic cigarette nicotine concentrations		
			0 mg/ml	8 mg/ml	16 mg/ml
*Rapid Information Processing Task*					
Digit rate	4.59	5.72	−2.90	2.69	5.68
Reaction time on correct trials	−6.94	0.13	20.13	−11.00	−6.88
Proportion correct	0.04	0.05	0.02	0.00	0.04
Commission errors	0.00	0.00	0.00	0.00	0.00
*Digit Symbol Substitution Task*					
Total trials completed	−1.34	**4.13** ^*∗∗*^	−1.25	**4.50** ^*∗∗*^	2.38
Percentage trials correct	0.00	0.01	0.01	0.04	0.01
*Visual Analog Scale-Smoking Effects *					
Confused	−0.09	2.38	0.88	0.25	1.88
Dizzy	0.41	11.13	−1.88	0.75	1.13
Headache	4.63	−2.75	−3.25	−1.50	−1.25
Heart pounding	0.22	1.45	0.38	0.00	1.01
Lightheaded	0.03	**18.88** ^*∗∗∗*^	0.75	2.75	0.25
Nausea	−0.47	0.38	0.00	0.00	0.00
Nervous	1.09	1.75	0.25	−1.63	−2.50
Salivation	−0.03	4.50	−1.25	1.00	5.50
Sweaty	0.03	0.00	−0.13	0.00	0.00
Weak	1.78	−2.38	−1.50	−1.00	0.00
*Minnesota Nicotine Withdrawal Scale*					
Angry, irritable, or frustrated	**0.72** ^*∗∗∗*^	−0.63	0.00	−0.25	−0.63
Anxious or nervous	0.13	0.00	0.00	−0.13	0.00
Depressed mood or sad	**0.19** ^*∗*^	−0.13	0.00	−0.13	−0.13
Desire or craving to smoke	**1.09** ^*∗∗∗*^	−**2.25**^*∗∗∗*^	−0.75	−0.63	−0.88
Difficulty concentrating	**0.50** ^*∗*^	−0.38	−0.38	−0.38	−0.13
Increased appetite, hungry, or weight gain	**0.28** ^*∗*^	−0.50	−0.25	0.00	−0.13
Restless	**0.41** ^*∗*^	−0.50	0.00	0.00	−0.25
Impatient	**0.69** ^*∗∗*^	−0.88	−0.13	−0.25	−0.25
Constipated	0.06	0.00	0.00	0.00	0.00
Dizziness	0.00	0.38	0.00	0.13	0.13
Coughing	−0.03	0.38	0.00	0.25	0.50
Nauseous	−0.03	0.00	0.00	0.00	0.00
Sore throat	0.00	0.25	0.13	0.25	0.38
Total score	**4.00** ^*∗∗∗*^	−4.25	−1.38	−1.13	−1.38
*Questionnaire of Smoking Urges-Brief*					
I have a desire for a cigarette right now	**22.78** ^*∗∗∗*^	−**51.25**^*∗∗∗*^	−6.50	−8.63	−10.00
Nothing would be better than smoking a cigarette right now	**19.09** ^*∗∗*^	−31.38	−12.25	−4.50	−14.13
If it were possible I would probably smoke now	**25.31** ^*∗∗∗*^	−**64.00**^*∗∗∗*^	−13.50	−15.13	−13.38
I could control things better right now if I could smoke	**9.38** ^*∗*^	−19.38	−6.63	−13.38	−0.50
All I want right now is a cigarette	**19.63** ^*∗∗*^	−**33.86**^*∗∗∗*^	−6.25	−14.88	−10.25
I have an urge for a cigarette	**25.88** ^*∗∗∗*^	−**52.25**^*∗∗∗*^	−7.75	−14.38	−15.50
A cigarette would taste good now	**20.38** ^*∗∗∗*^	−**44.00**^*∗∗∗*^	−8.63	−14.88	−12.38
I would do almost anything for a cigarette now	**14.94** ^*∗∗*^	−27.13	−1.13	−8.63	−6.38
Smoking would make me less depressed	8.84	−5.75	0.75	−2.00	−2.50
I am going to smoke as soon as possible	**24.84** ^*∗∗*^	−**47.50**^*∗∗∗*^	−6.00	−17.50	−14.8
*Heart rate and blood pressure *					
Heart rate	−**8.59**^*∗∗*^	**16.38** ^*∗∗∗*^	3.50	4.50	0.63
Mean arterial pressure	−3.61	6.04	1.71	2.04	0.96

All values are least-square means estimates from mixed models. Baseline versus 24-Dep = mean change between presmoking during baseline sessions and prior to OB or EC use during deprivation sessions. Own brand and electronic cigarette nicotine concentrations columns represent mean changes between pre- and postuse of OB or EC during deprivation sessions. _ _^*∗*^*p* ≤ .05, ^*∗∗*^*p* ≤ .01, and ^*∗∗∗*^*p* ≤ .001.
